# Psycho-oncology in Australia: a descriptive review

**DOI:** 10.1186/s13030-017-0100-1

**Published:** 2017-12-15

**Authors:** P. Butow, H. Dhillon, J. Shaw, M. Price

**Affiliations:** Psycho-Oncology Co-operative Research Group (PoCoG), Lifehouse level 6-North, C39Z, School of Psychology, University of Sydney, Sydney, NSW Australia

## Abstract

Australia has a thriving Psycho-Oncology research and clinical community. In this article, the Australian health system in which Psycho-Oncology is embedded is described. Clinical Psycho-Oncology services are outlined, in terms of their composition, processes and reach. The development of the internationally ground-breaking Australian Psychosocial guidelines for the care of adults with cancer is described. Two large Psycho-Oncology organisations which are strongly linked to mainstream Oncology organisations are discussed: the Australian Psycho-Oncology Society (OzPos, a primarily clinician-led and focused organisation) and the Psycho-Oncology Co-operative Research Group (PoCoG, a national cancer clinical trial group). OzPos is a special interest group within the Clinical Oncology Society of Australia, while PoCoG is one of 14 cancer clinical trial groups funded by the national government. It is these strong connections with major multidisciplinary cancer organisations, and a culture of collaboration and co-operation, that have made Psycho-Oncology grow and thrive in Australia. Examples of large collaborative programs of Psycho-Oncology research are provided, as well as the mechanisms used to achieve these outcomes.

## Background

Australia’s health system is a mixture of public and private health care. There is a strong national health scheme, called Medicare, which provides free general practice and hospital services for all citizens, although many health practitioners charge a “gap” fee - an amount over and above that provided by Medicare. In the past few years there has been a strong national emphasis on better access to mental healthcare (http://www.health.gov.au/internet/main/publishing.nsf/content/mental-ba-fact-pat), resulting in the ability of general practitioners (GPs) to write a mental health plan providing up to six free mental health consultations with registered practitioners such as Clinical Psychologists. Despite these services, there are strong incentives through the tax system for those with middle to high incomes to have private health insurance, covering varying degrees of health care.

Psycho-Oncology services are provided for free in larger public hospitals, sometimes through general hospital liaison services, sometimes through teams or personnel specific to the Oncology Department. Teams are comprised of social workers and psychologists, sometimes with a psychiatrist(s). Many of these services have long waiting lists. They vary in the degree to which they are incorporated into general oncology care, and in the power and autonomy they have to plan and direct their services. Most private hospitals and private medical practictioners’ rooms do not have psycho-oncology services provided, although referral can be arranged to private practitioners. In all Oncology services, the whole team, particularly nurse specialists and care co-ordinators, are tasked with providing psychosocial care to their patients.

There are numerous interwoven cancer organisations in Australia, which have been involved in the development of Psycho-Oncology. We refer to many of them below. For ease of understanding, we have included a diagram (Table 1), which outlines their functions and relationships. Psycho-Oncology and Palliative Care organisations have grown up somewhat independently, although they are clearly inter-related. Psycho-Oncology is seen to span the whole cancer trajectory, from high risk to diagnosis, to survivorship and/or death, while Palliative Care focuses more on those with incurable disease. Many individual researchers and clinicians are members of multiple organisations that address these two foci, and collaborations between psycho-oncology and palliative care researchers are very common.

**Table 1:** Cancer organisations in Australia

	National	State
Government	Department of Human Services: Medicare	State Health Departments
	Responsible for the development of service delivery policy, and provide access to health payments and services. Medicare provides access to a range of medical services, lower cost prescriptions and free care as a public patient in a public hospital.	Plans and provides health care, funding hospitals, clinics etc.
	National Health and Medical Research Council (NHMRC)	
	NHMRC aims to promote the development and maintenance of public and individual health standards. NHMRC provides national research funding for medical/health research, and provides advice to the Australian Government on health matters.	
	Cancer Australia	Cancer Institute NSW (CINSW)
	Cancer Australia is a national government body which aims to reduce the impact of cancer, address disparities and improve outcomes for people affected by cancer by leading and coordinating national, evidence-based interventions across the continuum of care. Funds research and develops resources.	NSW's government cancer control agency, established under the Cancer Institute NSW (2003) Act to lessen the impact of cancer across the State. Funds research and sets standards.
		The Victorian Cancer Agency (VCA)
		State body stablished to sustain and enhance Victoria’s excellent track record in cancer research. Funds research.
Clinical Professional Groups	Clinical Oncology Society of Australia: COSA	
	COSA is the peak national body representing health professionals from all disciplines whose work involves the care of cancer patients.	
	Single professional bodies. For example: Palliative Care Australia (PCA) PCA is the national peak body for palliative care. Australian Psycho-Oncology Group (OZPOS) OzPos is the national peak body for Psycho-Oncology.	Single professional bodies. For example: Palliative Care NSW, Victoria etc. State bodies for palliative care
Charity	Cancer Council Australia (CCA) The Peak body of the Cancer Councils, which are charity-based. CCA aims to promote cancer control at the national level.	Cancer Council NSW, Victoria etc. Charity-based cancer organisations to improve outcomes for cancer patients through research, advocacy and support services.
Clinical Trial groups	14 Cancer Clinical Trial Groups, funded by Cancer Australia, and under the umbrella of COSA, including: 1) Tumour specific trial groups, e.g.: Australian and New Zealand Breast Cancer Trials Group: ANZBCTG; Australia Lung cancer Trials Group: ALTG; Australian and New Zealand Gynaecological Oncology Group: ANGOG. 1) Treatment specific trial groups, e.g.: Trans Tasman Radiation Oncology Group: TROG; Palliative Care Clinical Studies Calloborative: PaCCSC; Psycho-Oncology Co-operative Research Group: PoCoG. Primary Care Collaborative Cancer Clinical Trials Group: PC4	There are also some state-based clinical trial groups, funded by CINSW or grants. E.g.: Improving Palliative Care through Clinical Trials (ImPACCT).

During the early 2000s, led by the National Breast Cancer Centre (NBOCC) (now incorporated into Cancer Australia, a national government body), Australia led the world in creating comprehensive psychosocial guidelines for the care of adults with cancer. This was a collaborative exercise involving many of the leaders in Psycho-Oncology, and provided a blue-print and advocacy tool for psychosocial care. The guidelines were approved by the National Health and Medical Research Council (NHMRC) of Australia, and endorsed by many professional groups. Since then, the psychosocial guidelines have undergone some review and revision, most recently with the addition of separate chapters on suffering, and fear of cancer recurrence. See https://canceraustralia.gov.au/clinical-best-practice/psychosocial-care/psychosocial-guidelines.

In 1996 a group of Psycho-Oncology researchers and clinicians formed a craft group within the Clinical Oncology Society of Australia (COSA), later incorporated as a company limited by guarantee as the Australian Psycho-Oncology Society (OzPOS), with a mission to :

- promote the psychosocial care of cancer patients and their families and carers
- enhance the capacity of health professionals to deliver optimal psychosocial care
- promote the timely translation of research into clinical practice.

Establishing a psycho-oncology craft group within the peak national multi-disciplinary cancer professional organisation has ensured our professional group a voice in policy and professional advocacy at the highest levels. Our involvement within COSA has resulted in the incorporation of psycho-social concerns amongst people living with cancer into all relevant position statements and in discussions with government and non-government stakeholders. COSA’s strong relationship with Cancer Council Australia, NBOCC and later Cancer Australia contributed to the priority given to developing the psycho-social care guidelines for cancer patients referred to above. Psycho-oncology involvement in COSA has also enabled us to highlight the importance of regularly monitoring the wellbeing of the oncology workforce through professional burnout surveys. More recently, we have contributed to the development of cancer survivorship care priorities and models of care to ensure the psychological wellbeing of cancer survivors into the future.

In individual Australian states, psycho-oncologists have informal networks. For example, in NSW, there is a network of Psycho-Oncology clinicians who meet monthly to exchange case studies, listen to invited speakers, and discuss professional issues. In Victoria, there is a Psycho-Oncology group allied to the Cancer Council of Victoria (a charity-based organisation) which advocates for psychosocial oncology services and creates information and resources for patients and families.

Funding for Psycho-Oncology research comes from a variety of sources, the largest being the National Health and Medical Research Council (NHMRC) which funds all health and medical research. Cancer Australia has created a collaborative research funding scheme (the Priority-driven Collaborative Cancer Research Scheme (PdCCRS) working with other funding partners such as the state cancer councils, Beyond Blue (a government depression organisation) and tumour specific foundations, such as Prostate Australia. In addition, there are state-specific funding mechanisms. For example, the Cancer Institute NSW (CINSW), a state government body, provides a significant amount of programmatic and project grant funding, much of it focused on implementation studies. Thus Psycho-Oncology researchers in many instances compete with all health and medical researchers, or more specifically with all cancer researchers, when applying for funds.

Investigator-driven clinical research in Australia has traditionally been led by clinical trial groups which are tumour specific (such as the Australian and New Zealand Breast Cancer Trials Group, ANZBCTG), or discipline-specific (such as the Trans Tasmanian Radiation Oncology Group, TROG). In 2005, the Psycho-oncology Co-operative Research Group (PoCoG), a clinical trial group specific to Psycho-Oncology, was formed in NSW when a funding opportunity became available through the Cancer Institute of New South Wales (CI NSW), responding to the recognised need to develop the capacity and co-ordinated collaboration to conduct large-scale, multi-centre psycho-oncology and supportive care research. Subsequently PoCoG received national funding for clinical trials infrastructure through the federal Department of Health and Cancer Australia, cementing its national presence in cancer clinical research. See http://www.pocog.org.au.

PoCoG aims to improve outcomes for people affected by cancer by developing and facilitating high quality, collaborative and clinically relevant research that focuses on interventions and services to optimise psychosocial and supportive care, with a focus on developing national research capacity and collaborations to conduct large-scale, multi-centre research of clinical relevance and importance, which would be difficult for a single team to undertake. In practice, PoCoG supports studies which target a psychosocial, behavioural or supportive care issue, address an issue that is amenable to intervention, are collaborative in nature, endeavour to address a research or clinical practice gap, engage populations typically underrepresented in clinical trials, have a strong rationale and methodology and have a Principal Investigator who is committed to obtaining funding for the study. This is achieved by providing expert advice, resources and support to researchers and health professionals to increase collaboration and innovation in psycho-oncology research and by bringing together researchers, clinicians, health care professionals and consumers to share ideas and form new collaborations and networks, and by encouraging and collaborating with other cooperative cancer trials groups.

Now more than 10 years old, PoCoG is an organisation of over 1500 researchers, allied health professionals, doctors, nurses, psychologists, and other health professionals across Australia and New Zealand who share a common mission of improving the emotional support and psychological care of people affected by cancer. Listed below are the structures and functions of PoCoG, with each bolded for ease of reference. Leadership is provided by the **PoCoG Chair,** the champion and mentor of the research group, and an adviser to the governing bodies. A small **Management Team** is responsible for the day to day management of PoCoG and for implementing the policies, procedures and strategic plan. The larger **Scientific Advisory Committee** (SAC) is responsible for determining the strategic direction and research priorities of PoCoG, is multidisciplinary and includes members with expertise in qualitative and quantitative research, statistics and/or diverse clinical roles. The **Joint Community Advisory Group** (JCAG) is a joint initiative between PoCoG and the Primary Care Collaborative Cancer Clinical Trials Group (PC4), formed to promote consumer participation in the groups’ activities, and functions as an advisory group to both PoCoG and PC4 as a sub-committee of the Scientific Advisory Committee of both groups. JCAG activities include reviewing research concepts from the community's perspective including the validity of the overall research question, facilitating links with members and advocating participation in research and clinical trials to the wider community. There are a number of PoCoG **Special Interest Groups,** including an Early Career Research group and a Clinicians’ Research group, proposed and led by PoCoG members, and supported by the PoCoG Executive Office.

In addition to resources made available via the website (e.g. email forums, upcoming events, research news items, online seminars), there are a number of core activities which mark the PoCoG year. **Concept Development Workshops** (CDWs) are a significant part of our groups’ efforts to foster development of large-scale, multicenter studies of clinical relevance and importance in psycho-oncology, and to educate and mentor early career researchers. These workshops provide a supportive environment for intensive input on study design and methods from scientific committee members and colleagues with expertise in biostatistics, health economics, and quality of life measurement, in addition to consumer input from members of JCAG. The **Scientific Advisory Committee meetings** are where there is ongoing review of PoCoG studies to ensure scientific rigour and maintenance of research quality standards. The development and use of **Standard Operating Procedures** are an integral part of quality assurance processes and provide written instructions on how to perform a task or process appropriately and consistently, and include study management processes, development of a study protocol, statistical analysis plan, ethics, data and document management, and study closeout.

The value of PoCoG is perhaps most clearly demonstrated in the Conquer Fear program of work. With fear of cancer recurrence (FCR) consistently reported by patients as a high unmet need [1], and no one specific intervention available for management [2], psychosocial clinicians from PoCoG collaborated to develop a novel 5-session manualised therapist-delivered psychological intervention specifically targeting high fear of recurrence in cancer survivors from 2008–2010. The intervention was based on the Common Sense Model of Illness, Self-Regulatory Executive Function Model (S-REF) and Relational Frame Theory and specialist psycho-oncology clinician expertise [3]. Following promising pilot testing [4], a multi-site, parallel randomised controlled trial led by PoCoG was planned. The protocol [5], guided by the PoCoG Standard Operating Procedures, was reviewed by the PoCoG Scientific Advisory Committee, commenced in 2012 and has recently been completed. The feasibility of this work was greatly enhanced by the support, engagement and commitment of 26 experienced psycho-oncology therapists employed across 15 Australian sites, to deliver of the study intervention. These clinicians were recruited via three Australian health professional networks: PoCoG, OZPOS, and Psychologists in Oncology. The PoCoG statistician provided invaluable statistical input into the design, data management and analysis plan for the study, while the PoCoG Management Team provided significant support in project management and ethical processes.

Another example of the key influence and outcome of PoCoG is the development of a clinical pathway for anxiety and depression. PoCoG identified the lack of a clinical pathway as a key barrier to implementation of routine screening and management of anxiety and depression in Australia. It brought together key leaders in the Australian psycho-oncology community and over the ensuing five years developed an evidence- and consensus-based clinical pathway for the screening, assessment and management of anxiety and depression in adults with cancer, specifically suited for the Australian health services system [6]. Instrumental in this work was using the PoCoG network of multi-disciplinary clinician members who provide cancer care (medical, surgical, primary care, nurses as well as psychosocial clinicians) across a range of settings in Australia, including rural and regional, public and private health care facilities. Again, using the PoCoG research network, a collaboration led by PoCoG has been recently funded to conduct a cluster randomized controlled trial to implement the pathways into standard care. Commencing in 2015 with the development of resources to support implementation, the trial will commence in late 2017.

A third example of successful PoCoG collaboration is the Rekindle trial. People living with a cancer diagnosis and their partners are known to experience substantial deterioration in their sexual function and well being, causing distress that can last many years after their cancer treatment has been completed. A group of University of Sydney researchers, in partnership with Cancer Council NSW, developed an online intervention to address the sexual concerns of cancer survivors across the full spectrum of the disease. In order to successfully implement and complete a phase II feasibility study [7], PoCoG established strong collaborations with most of the tumour specific cancer clinical trials groups such as the Australasian Lung Cancer Trials Group (ALTG) and Australasian Gastro-Intestinal Trials Group (AGITG), who supported and promoted the trial to their members, encouraging referral of patients into the study. Plans for a future phase III trial will continue and strengthen the relationship between our craft group and these disease group with access to substantial numbers of healthcare professionals supportive of research and working at the clinical interface. Successful recruitment to large scale clinical trials requires collaboration across the country.

There is a strong push in Australia to collaborate and work with other Psycho-Oncology groups internationally. PoCoG has strong links with international organisations such as the International Psycho-Oncology Society (IPOS) and the Asian-Pacific Psycho-Oncology Network (APPON). PoCoG is a member of the Federation of Psycho-Oncology national societies within IPOS, and has shared some resources with IPOS. Several leaders in PoCoG have sat or are sitting on the Board of IPOS while several PoCoG early careers researchers have formed and lead the IPOS Early Career Professionals in Psycho-Oncology group. Prof Butow (Chair of PoCoG) has presented several times at APPON, and sat on the APPON conference organising committee for 2016.

## Conclusions

In summary, Psycho-Oncology in Australia has benefited from strong links with the national clinical oncology body (COSA), other cancer clinical trial groups and international organisations such as IPOS and APPON, a collaborative culture, and the formation of a strong national research body (PoCoG). We look forward to working with colleagues in both Australia and internationally, in the decades to come.
